# *In silico* analysis and a comparative genomics approach to predict pathogenic trehalase genes in the complete genome of Antarctica Shigella sp. PAMC28760

**DOI:** 10.1080/21505594.2022.2117679

**Published:** 2022-08-30

**Authors:** Prasansah Shrestha, Jayram Karmacharya, So-Ra Han, Hyun Park, Tae-Jin Oh

**Affiliations:** a Department of Life Science and Biochemical Engineering, Graduate School, SunMoon University, Asan, Korea; b Division of Biotechnology, College of Life Sciences and Biotechnology, Korea University, Seoul, Korea; c Department of Life Science and Biochemical Engineering, SunMoon Univesity, Genome-based BioIT Convergence Institute, Asan, Korea; d Department of Pharmaceutical Engineering and Biotechnology, SunMoon University, Asan, Korea

**Keywords:** *Shigella* sp., trehalase, MP3, HMM, SVM, prokka

## Abstract

Although four *Shigella* species (*S. flexneri, S*. *sonnei*, *S. dysenteriae*, and *S. boydii*) have been reported, *S*. sp. PAMC 28760, an Antarctica isolate, is the only one with a complete genome deposited in NCBI database as an uncharacterized isolate. Because it is the world’s driest, windiest, and coldest continent, Antarctica provides an unfavourable environment for microorganisms. Computational analysis of genomic sequences of four *Shigella* species and our uncategorized Antarctica isolates *Shigella* sp. PAMC28760 was performed using MP3 (offline version) program to predict trehalase encoding genes as a pathogenic or non-pathogenic form. Additionally, we employed RAST and Prokka (offline version) annotation programs to determine locations of periplasmic (*treA*) and cytoplasmic (*treF*) trehalase genes in studied genomes. Our results showed that only 56 out of 134 *Shigella* strains had two different trehalase genes (*treF* and *treA*). It was revealed that the *treF* gene tends to be prevalent in *Shigella* species. In addition, both *treA* and *treF* genes were present in our strain *S*. sp. PAMC28760. The main objective of this study was to predict the prevalence of two different trehalase genes (*treF* and *treA*) in the complete genome of *Shigella* sp. PAMC28760 and other complete genomes of *Shigella* species. Till date, it is the first study to show that two types of trehalase genes are involved in *Shigella* species, which could offer insight on how the bacteria use accessible carbohydrate like glucose produced from the trehalose degradation pathway, and importance of periplasmic trehalase involvement in bacterial virulence.

## Introduction

*Shigella* is a Gram-negative bacterium that is genetically related to *Escherichia coli* [1]. It is a facultative anaerobe and a non-spore former. It belongs to non-motile and rod-shaped bacteria. *Shigella* are among common causes of diarrhoea worldwide. *Shigella* infection is one of the top four infections among African and South Asian children [2]. Based on its serological features, *Shigella* genus can be differentiated into four species: *S. dysenteriae* (serogroup A), *S. flexneri* (serogroups B), *S. boydii* (serogroups C), and *S. sonnei* (serogroup D). *Shigella* species has a highly immunogenic O-antigen made of many oligosaccharides unit (O) repeats with a wide range of sugar components, number of repeats, arrangements, and linkages. Each *Shigella* species can be further differentiated into several serotypes based on O-antigen on its lipopolysaccharide layer: *S. dysenteriae* having 15 serotypes, *S. flexneri* having 6 serotypes with 15 subtypes, *S. boydii* having 18 serotypes, and *S. sonnei* having only 1 serotype [3–5]. Although serogroups A, B, and C are physiologically identical, due to its positive beta-D-galactosidase and ornithine decarboxylase activity, *S. sonnei* is distinguished as a single serogroup D [6]. A previous study has reported that 60% of all infections worldwide are caused by *S. flexneri*. Thus, *S. flexneri* has been intensively studied, which has enhanced our understanding of *Shigella* pathophysiology and the underlying “host-pathogen” communication [7]. *S*. sp. PAMC28760 is a lichen-associated polar bacteria isolated from Antarctica. It has been deposited in the NCBI (National Center for Biotechnology Information) database (https://www.ncbi.nlm.nih.gov/) as an uncharacterized organism. Antarctica is a geographical mass covered with up to 13000 feet of ice and bare rock, with small mosses and lichens being its primary vegetation [8].

Various microorganisms remain unknown in such a harsh environment since they have developed specific adaption abilities towards a wide range of extreme conditions to thrive in such habitat [9]. Generally, *Shigella* species can grow in a temperature range from (6–8) °C to (45–47) °C [10]. However, temperatures about 65 °C cause their rapid inactivation. Some *Shigella* species can survive for long durations when they are frozen at −20 °C or refrigerated at 4 °C [11,12]. Bacteria have developed a wide range of coping mechanisms to endure adverse environments such as food deprivation, biochemical and biological changes, and extreme temperatures. Temperature is one of the most crucial elements that can influence microbial protein expression. According to previous studies, expression levels of outer membrane proteins were analysed using proteome profiles of *S. flexneri* cells grown at 37, 38.5, and 40° C. Pathogens might use the overexpression of specific proteins (18.4, 25.6, and 57.0 kDa) to govern the expression of virulence-related proteins when cells were exposed to higher temperatures [13]. Moreover, cold-adapted enzymes from organisms living in polar regions, deep oceans, and high altitudes have several benefits, they have been increasingly analysed in recent years.

Trehalose is also essential to organisms as a survival mechanism in a stress environment because of its unique physiochemical properties, which allow it to protect cell integrity against a different environmental damage and nutritional limitations [14]. Also, trehalose and its derivatives have also been found to possess crucial functions in the pathogenicity of a wide range of organisms, including bacteria (Gram-positive and Gram-negative) and plants [15] Also, trehalose metabolism could be employed as a target for novel pathogen-specific treatments. Trehalose is a disaccharide produced by various organisms. It can be degraded via several pathways. Among these pathways, the trehalose-6-phosphate pathway (TPP) is used by many bacteria to degrade trehalose. This pathway has been investigated under conditions of low osmolarity in both Gram-positive and Gram-negative bacteria [16,17]. It was reported in *E. coli* K-12 that under different osmolarity conditions, it may survive on trehalose as its sole carbon source and uses different pathways for its breakdown. Likewise, the external trehalose is hydrolysed by periplasmic trehalase (TreA) at high osmotic conditions. At that moment, the glucose PTS then transports the produced glucose molecules back into the cytoplasm [17,18]. During the transition between high and low osmolarity, a second trehalase, cytoplasmic trehalase (TreF), is active which removes the internal pool of trehalose as the cells alter their metabolism to low osmolarity. TreF’s low enzymatic activity is low enough not to interfere with trehalose biosynthesis during high osmolarity, but high enough to breakdown the accumulated trehalose during the return to normal conditions, when no more biosynthesis proceeds [19].

Several prokaryotes and eukaryotes can degrade trehalose to glucose through the enzyme trehalase [EC 3.2.1.28] [20,21]. It has been reported that *E. coli* has two trehalases, including cytoplasmic trehalase (TreF) and periplasmic trehalase (TreA). The periplasm is a small space between the outer and inner membranes of Gram-negative bacteria. Trehalases from *E. coli*, such as periplasmic TreA (Tre37A), have an extra C-terminal region, whereas TreF has an extended *N*-terminal region. Both enzymes are monomeric and have a 47% similarity [22]. Neutral trehalase (L72) is a protein found in *Klebsiella oxytoca* that has been linked to several functions, including energy sources and stress protection [23]. Experimental evidence of periplasmic *treA* gene in needed for optimal development of type 1 fimbriae for cell invasion and colonization in extraintestinal pathogenic *E. coli* (ExPEC) strain MT78 has been addressed in the previous study [24]. Similarly, in *Burkholderia pseudomallei*, a single trehalase-encoding gene, identical to *E. coli* TreA, which is involved in stress tolerance and virulence in mouse and insect infection models, plays a role in stress tolerance and virulence [25]. Despite its tiny size, the periplasm contains many important proteins required for a variety of physiological activities and bacterial survival under stress. Periplasmic proteins aid in the defence against different stresses, making it easier for bacteria such as *S. Typhimurium* to colonize the host [26]. However, there has been no complete analysis of the expression of many periplasmic proteins, especially periplasmic trehalase (TreA), in *Shigella* strains. The goal of this study was to determine the prevalence of two different trehalase genes (*treF* and *treA*) in 134 complete *Shigella* genomes, including lichen-associated *S*. sp. PAMC28760 isolated from the Antarctica region. Additionally, we would like to determine which trehalase genes (*treF* or *treA*) might contribute to virulence. It is thought that analysis of pathogenic and non-pathogenic trehalase might provide a new direction to understand bacterial pathogenic mechanism at the genetic level and to provide a new insight on drug development for the treatment of bacterial infections. The use of a bioinformatics tools such as MP3 can allow the study of virulence genes involved in respective strains without the need to perform hazardous laboratory experiments.

## Materials and methods

### Data sources

The complete genome and amino acid sequences of *Shigella* species were obtained from the NCBI database (https://www.ncbi.nlm.nih.gov/) [27]. A total of 134 *Shigella* strains deposited in NCBI by September 2021 were analysed, including our Antarctica isolate *S*. sp. PAMC28760, whose genome size was 4,558,287 bp [28].

### Phylogenetic tree construction and average nucleotide identity (ANI) analysis

To compare 16S rRNA sequences of *S*. sp. PAMC28760 with those in other complete genomes of *Shigella* strains (133 strains), phylogenetic analysis was performed using the ClustalW alignment tool and the Molecular Evolutionary Genetic Analysis (MEGA X) (https://www.megasoftware.net/) tools [29]. MEGAX was used to create the phylogenetic tree, which was built on a neighbour-joining tree method [30] and 1,000 bootstrap replications [31]. The online software Interactive Tree life (iTOL) v6 (https://itol.embl.de/) was used to analyse phylogenetic trees [32]. Orthologous Average Nucleotide Identity Software Tool (OAT) [33] was used to determine the average nucleotide identity (ANI) of 16S rRNA from closely related species acquired from EziBio Cloud (www.ezibiocloud.net) [34]. To determine if the strain PAMC28760 belonged to *Shigella* or *Escherichia*, EziBio cloud 16S rRNA sequencing was used. Secondary data was used to identify the cytoplasmic trehalase or periplasmic trehalase from the characterized strains *E. coli* strain K-12 substrain MG1655 (NC 000913.3) as a reference for the construction of a phylogenetic tree for trehalase genes (*treA* and *treF*) in those studied strains who possess both trehalase genes. NCBI, RAST, and Prokka were used to find the cytoplasmic and periplasmic genes. MUSCLE [35,36] was used to align amino acid sequences, and maximum-likelihood and neighbour-joining methods were used to build a phylogenetic tree.

### Comparative genomic analysis and, prediction of periplasmic trehalase and cytoplasmic trehalase

The prevalence of trehalase genes in the studied genome, as well as to predict pathogenic and non-pathogenic factors, were carried out using the MP3 (offline version) program (http://metagenomics.iiserb.ac.in/mp3/index.php) [37]. This program uses two modules including Support Vector Model (SVM) and Hidden Markov Model (HMM) to predict pathogenic and non-pathogenic proteins in the genome. Furthermore, Rapid Annotations utilizing Subsystems Technology (RAST, https://rast.nmpdr.org/rast.cgi) [38] and Prokka annotation (Prokka 1.14.6 offline version) [39] were used to locate predicted trehalase genes. CGView ServerBETA (www.cgview.ca) was used to better visualization of location predicted trehalase genes [40].

## Results and discussion

### Phylogenetic tree analysis of S. sp. PAMC28760

**Figure 1.**
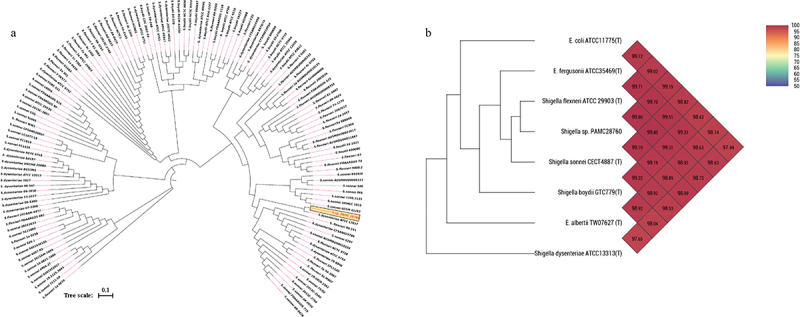
(**a**) Circular phylogenetic analysis of the complete genomes of *Shigella*: Phylogenetic tree showing the relationships of genomes of a total 134 *Shigella* strains including an Antarctica isolate *Shigella* sp. PAMC28760 (represented in red text), and their phylogenetic position. This analysis was prepared using MEGA X based on 16S rRNA sequences with neighbour-joining method with 1,000-replicate bootstrap. (**b**) Heatmap generated with OrthoANI values calculated using the OAT software to determine the close relationship of strain *S*. sp. PAMC28760 with *S. flexneri* ATCC29903(T), *S. sonnei* CECT4887(T), *E. coli* ATCC11775(T), *S. boydii* GTC779(T), *E. fergusonii* ATCC35469(T), *S. dysenteriae* ATCC13313(T), and *E. albertii* TW07627(T).

Phylogenomic analysis revealed that *S*. sp. PAMC28760 and *S. dysenteriae* ATCC12037 belonged to the same branch (Figure 1a). MEGA X program was used to construct phylogenetic tree to analyse their evolutionary history using the neighbour-joining method [41] with 1,000-replicate bootstrap. Furthermore, ANI value revealed that *S*. sp. PAMC28760 had a close relationship with strains *S. flexneri* ATCC29903(T) (99.80%), *S. sonnei* CECT4887(T) (99.70%), *E. coli* ATCC11775(T) and *S. boydii* GTC779(T) (99.19%), *E. fergusonii* ATCC35469(T) (99.70%), *S. dysenteriae* ATCC13313 (T) (98.99%), and *E. albertii* TW07627 (T) (98.89%) (Figure 1b). These results suggest that the *S*. sp. PAMC28760 strain is closely related to *Escherichia* strain as both belong to the same family *Enterobacteriaceae*.

### Trehalase gene and its phylogeny

**Figure 2.**
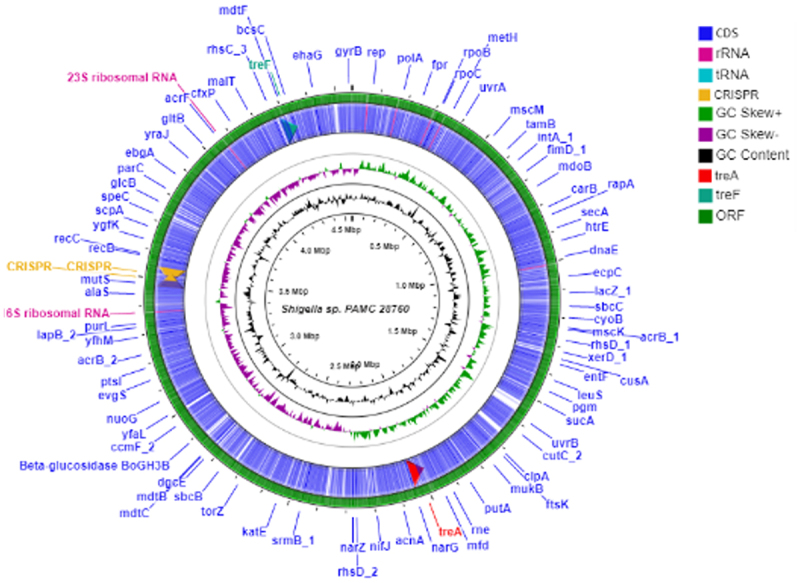
Circular genome comparison using CGView Server^BETA^ (http://cgview.Ca/) tool for the representation of genome and features of the *S*. sp. PAMC28760. The contents of the featured rings (starting with the outermost ring to the centre) are as follows. Ring 1, combined ORFs in forward and reverse strands; Ring 2, trehalose degradative genes, combined forward and reverse strand, and CDS (including tRNA and rRNA) in forward and reverse strands; Ring 3, GC skew plot, values above average are depicted in green, and below average in purple; Ring 4, GC content plot; and Ring 5, Sequence ruler.

**Figure 3.**
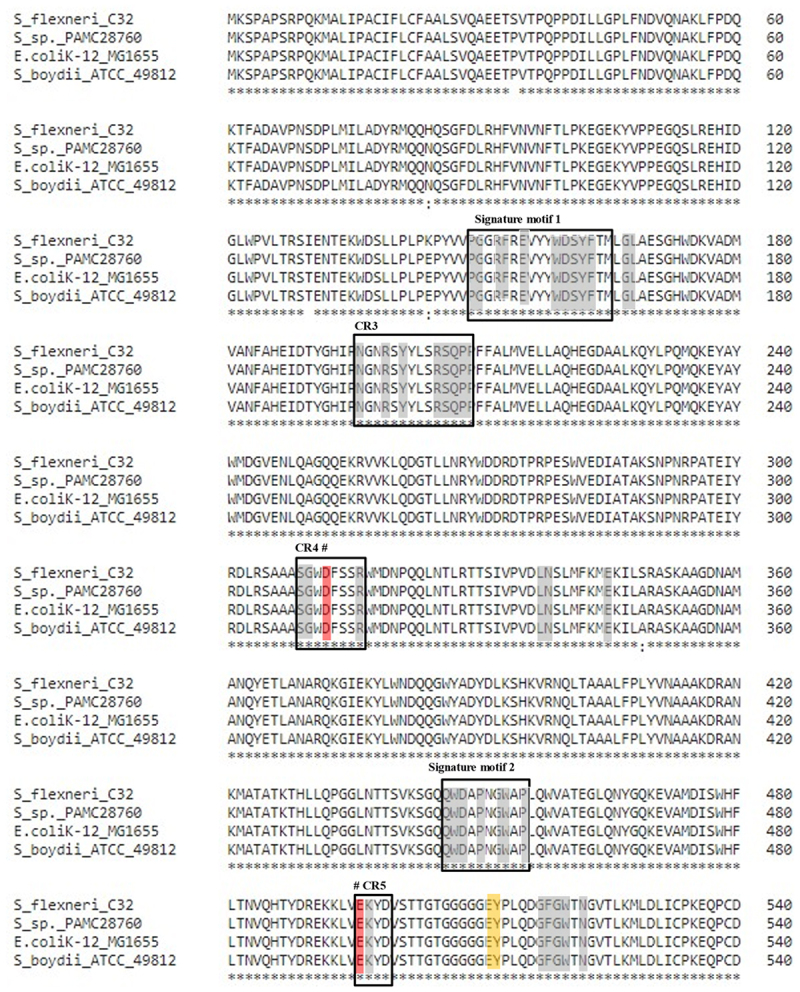
Cytoplasmic trehalase (TreF) amino acid sequence alignment with a characterized trehalase (TreF). TreF (GH37) from *E. coli* K-12 substr. MG1655, trehalase from *S. flexneri* C32, trehalase from *Shigella* sp. PAMC28760, and trehalase from *S. boydii* ATCC49812. The signature motif 1 and signature motif 2 represent two highly conserved sequence segments that belong to the GH37 family. The “#” symbol denotes the catalytic sites of Asp_312_ and Glu_496_. the three black boxes represent conserved regions (CR3–CR5).

**Figure 4.**
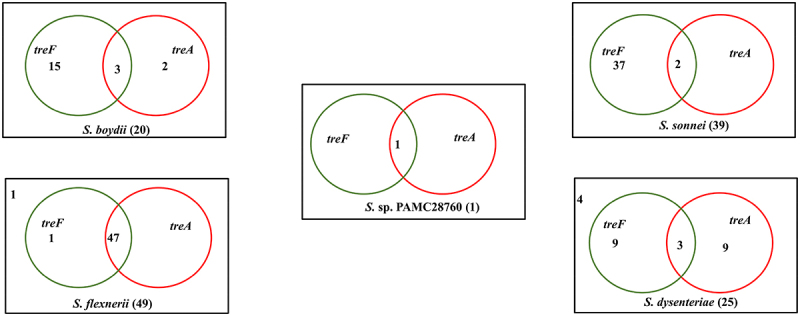
Venn diagram categorizes trehalase genes involved in the complete genomes of four *Shigella* species along with uncategorized *Shigella* sp. PAMC28760. Green circle represents the cytoplasmic trehalase (*treF*), whereas red circle represents the periplasmic trehalase (*treA*). The number outside the circles represents the absence of both trehalase genes.

When complete genomes of 134 *Shigella* strains including our strain PAMC28760 were studied, only 56 strains were found to have two types of trehalase (*treF* and *treA*) genes. Furthermore, we employed RAST annotation database and, Prokka annotation to differentiate cytoplasmic (*treF*) and periplasmic (*treA*) trehalase. In addition, the CGview online server (Figure 2) visualize the predicted trehalase genes in *S*. sp. PAMC28760. When we aligned them with characterized trehalase genes (*tre*F and *tre*A) of *E. coli* K-12 substrain MG655, *S*. sp. PAMC28760 was found to also encode the same genes involved in trehalose degradation (Figure 3). While 48, 47, and 47 of *S. flexneri*’s strains had *treF, treA*, and both *treF and treA* genes, respectively, 39, 2, and 2 of *S*. *sonnei’s* strains had *treF*, *treA*, and both *treF* and *treA* genes, respectively. In addition, of a total of 20 *S. boydii* strains, 18, 5, and 3 strains had *treF*, *treA*, and both *treF* and *treA* genes, respectively. For a total of 25 *S. dysenteriae* strains, 12,12, and 3 strains had *treF*, *treA*, and both *treF* and *treA* genes, respectively (Figure 4). Results showed that *S*. sp. PAMC28760 had both trehalase genes *treF* (cytoplasmic trehalase) and *treA* (periplasmic trehalase).

**Figure 5.**
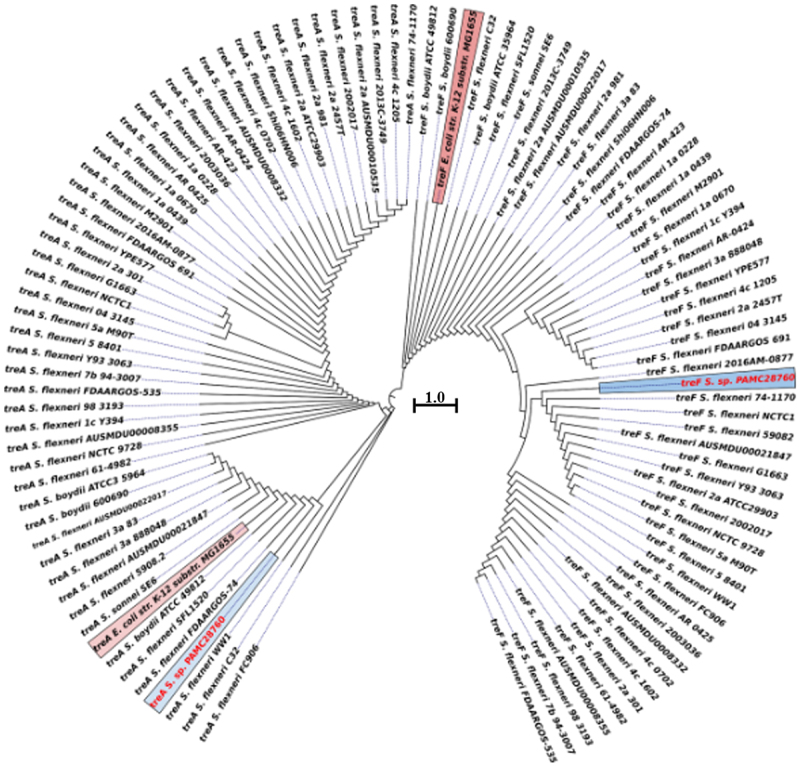
Circular phylogenetic tree based on trehalase genes (*treF*/*treA*) sequence in the complete genomes of *Shigella* strains with reference to the characterized trehalase of *E. coli* strain K-12 substrain MG165 using a neighbour-joining tree method with 1,000-replicate bootstrap. The pink highlighted boxes represent the characterized trehalase genes (*treF* and *treA*), whereas the red text indicates the strain (*Shigella* sp. PAMC28760) under study.

Phylogenetic tree analysis of trehalase genes (*treF* and *treA*) with a characterized *E. coli* K-12 substrain MG 1655 revealed that *treA* of *S*. sp. PAMC28760 and *E. coli* K-12 substrain MG1655 shared the same clade with 100% sequence identity, whereas *S*. sp. PAMC28760 did not share the same clade as *E. coli* K-12 substrain MG1655, although both shared 99.82% sequence identity (Figure 5). This shows that trehalase genes (*treA* and *treF*) of *S*. sp. PAMC28760 could be distinctly divided into two major clades. It was found that *treA* and *treF* genes from studied genome clustered together more closely with both genes of *S. flexneri*. The *treA* gene is clustered with *S. flexneri* FDAARGOS-74 and *S. flexneri* WW1 whereas *treF* is clustered with *S. flexneri* 2016AM–0877 and *S. flexneri* 74–1170.

These results suggest that *S*. sp. PAMC28760 might have a trehalose degradation pathway like that of *E. coli*. Also, it has been reported that TreA in *E. coli* is a trehalase found in the periplasmic area of cells that hydrolyzes trehalose glucose under high osmolarity, whereas TreF is a cytoplasmic isoform of TreA trehalase that plays important role in trehalose breakdown produced within bacterial cells under high osmolarity conditions [42,43]. Similarly, in the case of cytoplasmic trehalase (TreF), it becomes active during the transition between high and low osmolarity. TreF can deplete the internal trehalose pool as the cell metabolism shifts to a low osmolarity state. TreF has a low enzymatic activity that is low enough not to interfere with trehalose production under high osmolarity, but high enough to degrade the accumulated trehalose once the environment returns to normal [19].

### Trehalose degradative pathway

**Figure 6.**
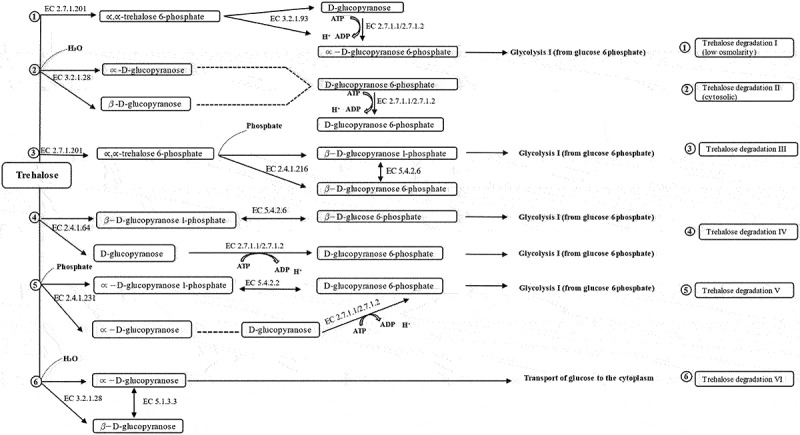
Trehalose degradative pathways. Six different trehalose degradative pathways are found in organisms (bacteria, fungi, yeast, Arthropoda, and plants). Among them, only two degradation pathways (Trehalose degradation pathway II (cytosolic) and VI (periplasmic)) are found in *Shigella* species.

**Figure 7.**
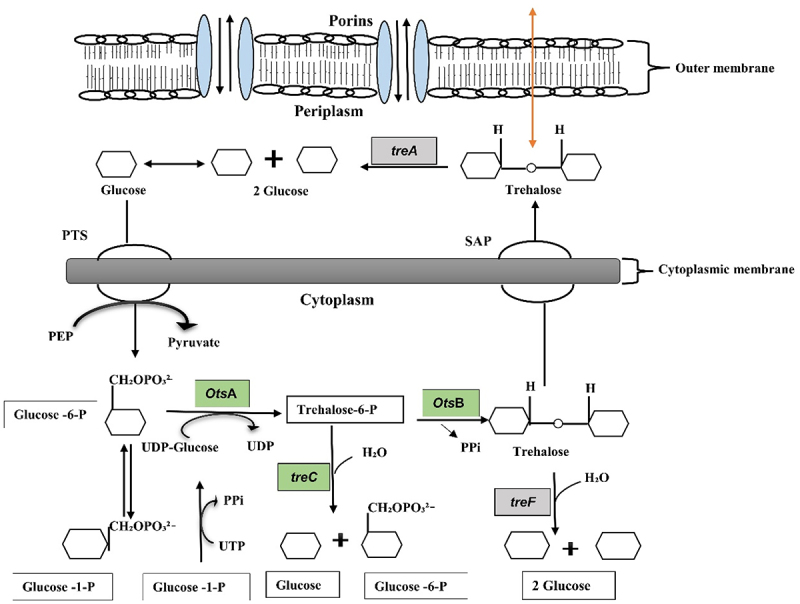
Schematic diagram of the trehalose metabolism pathway in Gram-negative bacteria is formulated from Kosciow et al., 2014 and Purvis et al., 2005. The green boxes represent the trehalose synthesis genes (*otsA*, trehalose-6-phosphate phosphatase; *otsB*, trehalose-6-phosphate synthase; and *treC*, trehalose-6-phosphate hydrolase), whereas grey boxes represent the trehalose degrading genes (*treA*, periplasmic trehalase; and *treF*, cytoplasmic trehalase). At cytoplasm, trehalose is degraded by cytoplasmic trehalase gene (*treF*). The plasma membrane, stretch-activated proteins (SAP) facilitate the exit of trehalose under hypotonic conditions to the periplasm where it further degraded by periplasmic trehalase gene (*treA*).

Six routes of trehalose degradation pathways (trehalose degradation I, II, III, IV, V, and VI) have been found in organisms depending on their subcellular locations. These pathways have been reported in the MetaCyc pathway database [44]. They are summarized in (Figure 6). Depending on the organism, trehalose might enter cells via a permease where it remains unmodified, or it gets transformed to phosphorylated trehalose 6-phosphate forms via a phosphotransferase system (PTS). Trehalose that cannot be modified might get degraded by a hydrolysing trehalase (EC 3.2.1.28) or might be split by trehalose phosphorylase (EC 2.4.1.64, and EC 2.4.1.231) (Figure 7). It was revealed that our Antarctica isolate *S*. sp. PAMC28760 had the trehalase gene based on the prediction of trehalose degradative pathway. The result is summarized in Figures 2 and 6. Trehalose is broken down into two molecules of glucose and water by the trehalase enzyme that utilizes glucose as a carbon source. Trehalase is classified into glucoside hydrolase (GH) families such as GH37, GH65, and GH15 in the CAZy (Carbohydrate-Active Enzyme) database (http://www.cazy.org/) [45]. The GH37 family possesses only trehalase enzymes, whereas GH65 and GH15 families possess other enzymes along with trehalase enzymes. In 2007, it was reported that *Mycobacterium* s*megmatis* and *Mycobacterium tuberculosis* possessed trehalase that belonged to the GH15 family [46].

Trehalase belonging to the GH37 family can hydrolyse a molecule of ∝,∝-trehalose into two molecules of glucose by inverting the anomeric orientation. Trehalase belonging to the GH37 family have been found in different species, including bacteria, fungi, yeasts, plants, insects, and vertebrates [22]. GH family has been divided into “clans” in the CAZy database, where enzymes are regarded to have a common evolutionary origin. Clan GH-G was ascribed to GH37 enzymes, while clan GH-L was ascribed to GH65 and GH15 enzymes. Although clans GH-G and GH-L share only a low amount of sequence homology, such finding is significant. GH37 trehalase has two catalytic residues, Asp and Glu, in their CDs (catalytic domains). Asp and Glu residues tend to be involved in the function of GH65 and GH15 trehalases. These amino acid residues are most likely to be involved in a common inverting mechanism during catalysis [47]. Structures of these trehalases are comprised of conserved regions (CRs), which include catalytic residues. These CRs can form active sites that usually have loops. CDs of GH enzymes contain well-known trehalase signature motifs, motif 1 (PGGRFXEXY[G/Y] D[S/T] Y] and motif 2 (QWD[Y/F]PN/Y) [G/A] W[P/A] P), whereas GH65 and GH15 trehalases do not [48,49]. Our Antarctica isolate *S*. sp. PAMC28760 possesses GH37 trehalase with two signature motifs (motifs 1 and 2) as well as highly conserved regions (CR3-CR5), which have also been found in *E. coli*. Further study confirms that *S*. sp. PAMC28760 possesses trehalase enzyme, a member of the GH37 CAZyme family (Figure 3). The Gram-positive bacteria like *Bacillus subtilis* (non-pathogenic) and *Clostridioidess difficile* (pathogenic) share a pathway in which exogenous trehalose can be imported by a PTS to produce glucose and glucose-6-phosphate via the phosphotreahalose TerA (analogous to the PTS-TreC system in pathogenic *E. coli*). Due to the acquisition of an additional cluster of trehalose metabolism genes, namely a second PTS that mediates high-efficiency trehalose uptake from the environment, epidemic *C. difficile* strains can also grow on low trehalose. By increasing toxin levels, both modified trehalose utilization systems contributed to the growth and toxicity of these epidemic *C. difficile* strains [49]. There have been no previous papers on the function of the trehalose degradation pathway in virulence in Antarctic isolates till date. However, in *Variovorax* sp. PAMC28711 [50], the presence of trehalose metabolic pathway was mentioned.

### Prediction of pathogenic and non-pathogenic proteins

MP3 (standalone program) can predict the presence of pathogenic and non-pathogenic proteins in a complete genome of a microbe based on two models, SVM and HMM, and their hybrids (integrated SVM and HMM models). To predict pathogenic and non-pathogenic trehalase, we retrieved complete genomes of 134 *Shigella* species (strains) from the NCBI database along with our *S*. sp. PAMC28760 isolates from Antarctica. Our strain *S*. sp. PAMC28760 showed pathogenic proteins of 1,136 (based on SVM model) out of 4329 total proteins (Table 1), with periplasmic trehalase as a pathogenic trehalase (data not shown). MP3 tool can be used to compare numbers of pathogenic proteins in healthy and infected samples by precisely identifying pathogenic protein fragments (based on amino acid composition and dipeptide composition) commonly found in metagenomic data without needing a time-consuming homology-based alignment [37]. In comparison with other publicly available bioinformatic tools, this program can predict pathogenic proteins with improved accuracy (95.06%), sensitivity (85.59%), and specificity (96.64%) as it employs both SVM and HMM models. Also, it is essential to analyse complete genome sequences of pathogenic and non-pathogenic bacteria of closely related species to determine if any significant genomic changes have occurred. It has been proposed that both pathogenic and non-pathogenic strains have virulence factors/genes. They can be distinguished based on gene content. When other genes suppress the virulence factors/genes, the bacterium becomes non-pathogenic. However, when suppressing genes are lost, a commensal can become pathogenic [51].

**Table 1.** MP3 prediction of the total proteins, pathogenic protein, and non-pathogenic proteins in all the complete genomes of *Shigella* strains including *Shigella* sp. PAMC28760, which is indicated as a asterisk symbol. Hybrid: predictions from both HMM and SVM models.

Strain	Total proteins	HMM	Hybrid	SVM	Strain	Total proteins	HMM	Hybrid	SVM	Strain	Total proteins	HMM	Hybrid	SVM
*Shigella flexneri*					*Shigell*					***Shigella boydii***				
S. flexneri C32	4746	367	1126	1259	S. sonnei 2015C_3566	4295	279	1002	1125	S. boydii 54_1621	3409	181	690	803
S. flexneri 1a 228	3973	254	843	955	S. sonnei 2015AM-1099	4318	281	992	1115	S. boydii 59_248	3958	254	855	967
S. flexneri 1a 439	4086	262	873	1001	S. sonnei AR_0426	4120	265	887	1004	S. boydii 83_578	3725	219	777	896
S.flexneri 1a 670	4067	276	880	997	S. sonnei ATCC 29,930	4140	273	929	1041	S. boydii ATCC 8700	3436	204	725	818
S. flexneri 2a 981	4056	257	873	993	S. sonnei FC 1428	3930	252	879	998	S. boydii ATCC 9210	3807	231	801	914
S. flexneri 2a 2457T	3827	236	805	923	S. sonnei FDAARGOS 715	4149	274	931	1061	S. boydii ATTC 35,964	4070	248	887	1004
S. flexneri 2a AUSMDU00010535	4043	269	892	1019	S. sonnei KCCM41282	4041	269	892	1006	S. boydii ATCC 49,812	4347	285	971	1090
S.flexneri 2a str 301	4313	260	835	959	S.sonnei 866	4086	274	919	1046	S. boydii ATCCBAA_1247	3723	228	783	905
S. flexneri 4c 702	3996	250	853	964	S. sonnei 53 G	4648	313	1119	1239	S. boydii CDC 3083_94	3909	252	854	970
S. flexneri 5a M90T	3972	260	863	984	S. sonnei 75_02	4583	319	1106	1231	S. boydii KCCM 41,690	3650	212	749	867
S. flexneri 64-5500	3981	250	870	981	S. sonnei FDAARGOS_524	4114	899	899	1023	S. boydii NCTC 9733	3611	240	793	885
S. flexneri 74_1170	4099	261	887	1015	S. sonnei Ss046	4056	282	903	1026	S. boydii NCTC 9850	3749	224	792	909
S. flexneri 2016AM_0877	4062	269	875	994	S. sonnei FORC_011	4499	306	1087	1218	S. boydii Sb 227	3819	227	805	924
S. flexneri 61_4982	3933	240	811	931	S. sonnei 2015C_3794	4218	272	987	1111	S. boydii 59_2708	3753	236	780	894
S. flexneri 2,002,017	4045	263	879	998	S. sonnei CFSAN030807	4316	288	1016	1142	S. boydii NCTC9353	3318	177	672	778
S. flexneri 2,003,036	3770	235	235	907	S. sonnei FC1653	3930	256	865	986	S. boydii 600,657	3702	240	888	777
S. flexneri AR_0424	4037	262	880	996	S. sonnei LC1477_18	4048	268	908	1034	S. boydii 600,080	3784	234	928	807
S. flexneri AR0423	3980	251	848	960	S. sonnei AUSMDU00008333	4184	272	938	1059	S. boydii 600,690	4023	267	965	807
S. flexneri FC906	3882	239	822	950	S. sonnei AR_0030	4319	277	956	1080	S. boydii 602,068	3777	245	796	903
S. flexneri G1663	3976	261	261	971	S. sonnei 2015C_3807	3857	274	840	950	S. boydii FDAARGOS_1139	3641	221	748	855
S. flexneri shi06HN006	3795	237	804	916	S. sonnei AUSMDU00010534	4165	280	921	1045					
S. flexneri Y 93-3063	4100	275	911	1027	S. sonnei FDAARGOS_90	4149	182	931	1061					
S. flexneri Y PE577	3807	239	802	915	S. sonnei 19.0821.348	4196	260	883	1006					
S. flexneri FDAARGOS_74	3925	262	847	967	S. sonnei 19.1125.3493	4097	260	862	983	**Strain**	**Total proteins**	**HMM**	**Hybrid**	**SVM**
S. flexneri 1c Y394	3922	258	834	951	S. sonnei 506	4505	295	982	1099	S.sp. PAMC 28,760*	4329	303	1006	1136
S. flexneri AR_0425	3937	259	848	961	S. sonnei 1205.3131	4201	267	887	1013					
S. flexneri 7b 94_3007	4117	273	900	1021	S. sonnei 6207	4260	269	909	1021					
S. flexneri NCTC 9728	3886	245	817	939	S. sonnei 6607	4112	262	876	993					
S. flexneri 98_3193	3665	216	765	880	S. sonnei 6904.27	4022	260	859	974					
S. flexneri AUSMDU00008355	3905	246	830	953	S. sonnei 7111.69	4168	262	873	999					
S. flexneri 89_141	3880	252	835	947	S. sonnei 3,123,885	3916	251	832	947					
S. flexneri 4c 1205	4676	295	1022	1160	S. sonnei 9,163,633	4165	261	872	1003					
S. flexneri 04-3145	3785	237	784	899	S. sonnei 401,930,105	4044	257	861	977					
S. flexneri NCTC1	3769	234	784	898	S. sonnei L4094	4127	266	886	1005					
S. flexneri SFL1520	3833	236	809	915	S. sonnei SE6-1	4262	325	960	1078					
S. flexneri 5str 8401	3838	244	807	919	S. sonnei UKMCC-1015	4146	268	874	986					
S.flexneri 2a ATCC 29,903	4117	253	895	1014	S. sonnei 401,952,027	4141	259	867	990					
S. flexneri 4c 1602	4169	276	924	1045	S. sonnei LC1477/18	4141	259	908	1034					
S. flexneri FDAARGOS_535	4059	270	892	1012	S. sonnei 893,916	3864	241	810	928					
S. flexneri AUSMDU00008332	4116	269	908	1033										
S. flexneri 3a 888,048	3611	227	746	860										
S. flexneri 2013C_3749	4024	261	878	1001										
S. flexneri 5908_2	3777	241	802	922										
S. flexneri FDAARGOS_691	3730	230	783	904										
S. flexneri M2901	4092	261	883	1006										
S. flexneri AUSMDU000021847	5533	348	1276	1442										
S. flexneri AUSMDU00022017	5494	343	1283	1434										
S. flexneri WW1	4413	348	1020	1140										
S. flexneri 83	5230	331	1225	1370										
S. dysenteriae ATCC9753	3944	243	854	971										
S. dysenteriae ATCC9754	2959	164	601	679										
S. dysenteriae ATCC12037	3831	249	830	946										
S. dysenteriae ATCC12039	3942	252	820	941										
S. dysenteriae ATCC49346	3689	229	786	898										
S. dysenteriae ATCC49347	3868	241	823	941										
S. dysenteriae BU53M1	3697	229	760	869										
S. dysenteriae CFSAN010954	2688	150	554	625										
S. dysenteriae CFSAN010956	4019	274	890	1014										
S. dysenteriae CFSAN029786	3917	262	829	946										
S. dysenteriae 07_3308	3274	175	614	721										
S. dysenteriae 08_3380	3518	213	700	812										
S. dysenteriae 53_3937	3310	179	628	736										
S. dysenteriae 69_3818	3462	207	688	790										
S. dysenteriae 1617	3140	176	625	756										
S. dysenteriae 2017C_4522	3621	216	759	866										
S. dysenteriae ATCC9752	3087	178	619	713										
S. dysenteriae ATCC13313	3474	208	686	792										
S. dysenteriae E670_74	4364	271	964	1105										
S. dysenteriae NCTC9718	3356	199	651	756										
S. dysenteriae Sd197	4294	222	695	804										
S. dysenteriae 80_547	3462	207	688	790										
S. dysenteriae ATCC9751	3062	182	642	734										
S. dysenteriae 79_8006	3698	228	760	874										
S. dysenteriae HNCMB20080	3296	179	610	719										

In addition, the detection of transposon mutants in extraintestinal pathogenic *E. coli* (ExPEC) that are defective in binding to non-phagocytic cells is an unexpected finding on the probable role of periplasmic trehalase (*treA*) in virulence [24]. Furthermore, while trehalase enzymes are known to have a role in virulence of some fungal species, the occurrence of multiple enzymes can inhibit their potential as an antifungal drug target. Because the trehalose pathway and its enzymes are not found in mammals (including humans), fungi-specific inhibitors of the trehalose pathway and their enzymes should be generally non-toxic to mammals [52,53]. Likewise, a previous study has reported that inactivating trehalose biosynthesis pathways does not reduce resistance to oxidative stress in many bacteria, but a periplasmic trehalase gene (*treA*) mutant in *Burkholderia pseudomallei* shows increased sensitivity to oxidative stress despite elevated trehalose levels in the mutant, which is expected to protect against this stress [25]. Another study also reported that validmycin A was ineffective against *Clostridioides difficile* TreA, whereas trehalose derivatives such as epimers containing hydroxyl groups (2- and 4-positions), and thiotrehalose derivatives showed promise as TreA inhibitors with a larger spectrum. The efficacy of these drugs in treating specific bacterial infections is currently being studied [54]. It has also been reported that the PTS route for trehalose uptake (trehalose degradation I, low osmolarity) is inhibited when the osmolarity is high. Thus, trehalase (TreA) in the periplasm can allow cells to utilize trehalose at a high osmolarity by breaking it down into glucose molecules, which can be subsequently transported by phosphotransferase mediated system [55]. Genome of *Shigella* strains were analysed for pathogenic and non-pathogenic trehalase genes in this study for the first time. It is assumed that studying trehalase in one pathogenic bacterium like *Shigella* species could be important for further studies. Trehalase (TreA) from the pathogenic strain of extraintestinal *E. coli* known as MT78 has also been identified as a member of glycoside hydrolase 37 (GH37). Similarly, deletion of these genes in the meningoencephalitis-causing yeast *Crytococcus neoformans* resulted in severe defects in spore production, a decrease in spore germination, and an increase in the production of alternative development structures, which spores forms are plausible infectious particles [56]. Trehalose does not have to solely play a role in osmoregulation. According to Lee et al., it has stated that if glucose is present in the cytoplasm, molecules like trehalose are produced at levels approaching 400 mM in the cytoplasm [57]. Glycine betaine and L-proline often accumulate in the cytoplasm (around 700 and 400 mM, respectively) and can replace trehalose [58]. Many species utilize these osmolytes, which appear to be well-adapted to cellular functions. The electro-neutral solutes trehalose, glycine betaine, and L-proline, as well as potassium glutamate, have various chemical characteristics that may suit their functions in cell survival during osmotic shock.

## Conclusions

Although there are many studies on trehalase, it was not studied in *Shigella* species based on two different trehalase genes (*treF* and *treA*) and pathogenicity. Most *Shigella* species (*S. flexneri*, *S. boydii*, *S*. *dysenteriae*, and *S. sonnei*), as well as our strain *S*. sp. PAMC28760, have cytoplasmic trehalase, and all periplasmic trehalase predicted in the studied strains showed up as pathogenic proteins using MP3, RAST, and Prokka tools. Notably, *treF* was detected in all strains of *S. sonnei*, but *treA* was identified in only two strains. This sort of research on pathogenic and non-pathogenic trehalase could help researchers to elucidate how and why *Shigella* species have certain traits. Furthermore, before performing any kinds of wet lab work, these bioinformatics tools are important in determining the nature of proteins present in a complete genome of bacteria.

## Data Availability

Data used in this study are available from the corresponding author upon reasonable request.
